# Acceptance of a Web-Based Intervention in Individuals Who Committed Sexual Offenses Against Children: Cross-Sectional Study

**DOI:** 10.2196/48880

**Published:** 2024-01-26

**Authors:** Sonja Schröder, Claudia Buntrock, Louisa Neumann, Jürgen L Müller, Peter Fromberger

**Affiliations:** 1 Clinic for Psychiatry and Psychotherapy – Forensic Psychiatry University Medical Center Göttingen Göttingen Germany; 2 Institute of Social Medicine and Health Systems Research Otto von Guericke University Magdeburg Magdeburg Germany; 3 Clinic for Forensic Psychiatry and Psychotherapy KRH Psychiatry Wunstorf Wunstorf Germany

**Keywords:** mHealth, web-based intervention, acceptance, Unified Theory of Acceptance and Use of Technology, UTAUT, sexual offenses against children, child abuse, child pornography, children, sexual offense, cross-sectional study, community, anxiety, psychiatry

## Abstract

**Background:**

Individuals who have committed sexual offenses against children often have difficulties finding treatment, despite its potential effectiveness. Although the development of web-based interventions could enhance therapeutic supply, up to now the acceptance thereof among this target group is unknown.

**Objective:**

For the first time, this study assesses the acceptance of a web-based intervention among individuals who committed sexual offenses against children and analyzes variables that predict acceptance. Following the Unified Theory of Acceptance and Use of Technology (UTAUT), it is assumed that acceptance of web-based interventions in individuals who have committed sexual offenses against children follows the same mechanisms as for individuals in general psychiatry.

**Methods:**

This cross-sectional study is based on the data from an ongoing clinical trial (@myTabu) evaluating the effectiveness of a web-based intervention in individuals who committed sexual offenses against children (N=113). Acceptance level was measured using a questionnaire based on the UTAUT and modified for the target group. Furthermore, predictors of acceptance from the UTAUT (performance expectancy, effort expectancy, and social influence [SI]), attitudes toward web-based interventions, and internet anxiety were assessed at baseline.

**Results:**

Most participants (61.1%, 69/113), reported high acceptance, while 36.3% (41/113) of them indicated moderate acceptance, and 2.7% (3/113) of them expressed low acceptance. In a linear regression model, the predictors explained 41.2% of the variance (*F*_11,101_=9.055; *P*=.01). Attitudes toward web-based interventions (*B*=0.398, 95% CI 0.16-0.64; *P*=.001) and SI (*B*=0.183, 95% CI 0.03-0.38; *P*=.04) significantly predicted acceptance. Post hoc explorative analysis showed that the participants’ belief that people close to them would recommend the use of a web-based intervention is a predictor of acceptance. In contrast, the belief that their community supervisor would recommend the use thereof was not predictive in this respect.

**Conclusions:**

For the participants of this study, we identified high acceptance of web-based interventions for the majority of participants. SI and the participants’ attitudes toward web-based interventions were important in predicting acceptance.

**Trial Registration:**

German Clinical Trial Registration (DRKS, Deutsches Register Klinischer Studien) DRKS 00021256; https://drks.de/search/de/trial/DRKS00021256

## Introduction

### Background

Sexual abuse during childhood has disruptive short and long-term effects for children who are victims of such an offense [1,2] and the treatment of individuals who committed sexual offenses against children should be a major part of efforts to reduce the risk of recidivism. Despite findings that therapy can reduce the risk of recidivism [3], many individuals who committed sexual offenses against children struggle to find a therapist. Therapists often express a low willingness to work with individuals who are convicted of a sexual offense—especially with those who have a pedophilic disorder [4]. The result, at least in Germany, is that only limited therapeutic treatment is available [5]. Web-based interventions represent a possible enhancement in the therapeutic supply [6].

Web-based interventions can be advantageous in comparison to face-to-face (f2f) therapy for the users, as they can be anonymous, flexible in time and space, and can be cost-effective [6,7]. Anonymity could be especially advantageous, as individuals who committed sexual offenses against children can feel ashamed and guilty which may hinder the willingness to find a therapist. To date, only a few web-based interventions exist for individuals who have committed sexual offenses against children and the majority of them have not yet been evaluated [8]. In a placebo-controlled trial, Lätth et al [9], showed for the first time that a guided web-based intervention for individuals who consume child exploitation material can reduce the amount of time thus spent. In addition, the study showed that, as is the case in web-based interventions in general psychiatry [10], many persons who signed up for a web-based intervention did not participate by logging in or completing the therapeutic content [9]. Also, in f2f therapy for individuals who have committed offenses, roughly one-third of individuals do not complete therapy [11,12]. Up to now the variables that predict why and for how long individuals who have committed sexual offenses against children use web-based interventions are unknown. In general, a factor that is expected to predict whether someone uses web-based interventions in general psychiatry is acceptance [13,14]. Thus, this predictor might also be important in the treatment of individuals who committed sexual offenses against children.

To study acceptance and its predictors, research on web-based interventions for general mental health often uses the Unified Theory of Acceptance and Use of Technology (UTAUT) as a theoretical framework [15,16]. The UTAUT states that the use of a technology can be predicted by acceptance. Acceptance is thereby defined as the behavioral intention to use a technology. Further, 4 core predictors are assumed, which are performance expectancy (PE), effort expectancy (EE), social influence (SI), and facilitating conditions (FC). PE is related to whether or not the person believes that the web-based intervention can help him or her; EE is related to the perceived ease of use of the web-based intervention; SI is the perception of whether people close to him or her would recommend the use of the web-based intervention; and FC is related to the belief that there is an organizational and technical infrastructure that would help him or her in case of problems with the web-based intervention. According to the UTAUT, FC together with acceptance predict the use of technology. The other 3 variables, PE, EE, and SI, predict acceptance.

Although the UTAUT was first conceptualized to explain the use of technology in organizational settings, it has been generalized to many different fields including the use of technology for treatment in general psychiatry [16]. Philippi et al [16] conducted a secondary analysis in which they integrated the data of 1588 participants from 10 UTAUT studies. The original studies analyzed the participants’ acceptance and its predictors based on the UTAUT for web-based interventions, for example, for treating depression, chronic pain, or aftercare for inpatients. In the study by Philippi et al [16], the basic structure of the UTAUT with PE, EE, and SI predicting acceptance was replicated. PE was found to be the strongest predictor, in accordance with results from prior studies [15,17,18].

Gender, age, degree of voluntary use of technology, and experience with the technology were included next to predictors in the UTAUT as moderators [15]. The authors showed that the effect of PE was stronger for younger and male individuals; the effects of EE was stronger for older, female, and less experienced individuals; and the effect of SI was stronger for older, female, and less experienced individuals as well as under conditions of mandatory use [15]. In web-based interventions in general psychiatry, however, Philippi et al [16] could not replicate a moderating effect of age, gender, or experience on any predictor. Next to moderating effects, a direct effect on acceptance of participant age was analyzed. In some studies on web-based interventions in general psychiatry, it was found that a lower age predicted higher acceptance [19-21] whereas other studies found no effect [16,22].

In the field of web-based interventions in general psychiatry, the variables attitudes toward web-based interventions and internet anxiety were also integrated into the UTAUT to predict acceptance. Attitudes refers to the evaluative judgment of a web-based intervention, which can be expressed in attributes ranging for example, from pleasant to unpleasant or likable to dislikable [20,23]. Internet anxiety is the fear, distrust, or apprehension that is experienced when using the internet [16,24]. Attitudes and computer anxiety were removed from the final UTAUT model because the explorative power of the variable was captured by EE [15]. In recent studies, however, attitudes was found to be a strong predictor for acceptance [20,25,26]. Similarly, internet anxiety studies have shown that persons with lower internet anxiety have a higher acceptance for web-based interventions in general psychiatry [16,22,26].

### Objective

The goal of this study is to address the following research questions for individuals who committed sexual offenses against children, either by contact or noncontact offense (ie, child sexual exploitation material offenses): (1) how high is the acceptance of web-based interventions? (2) Which variables predict acceptance of web-based interventions?

**Figure 1:**
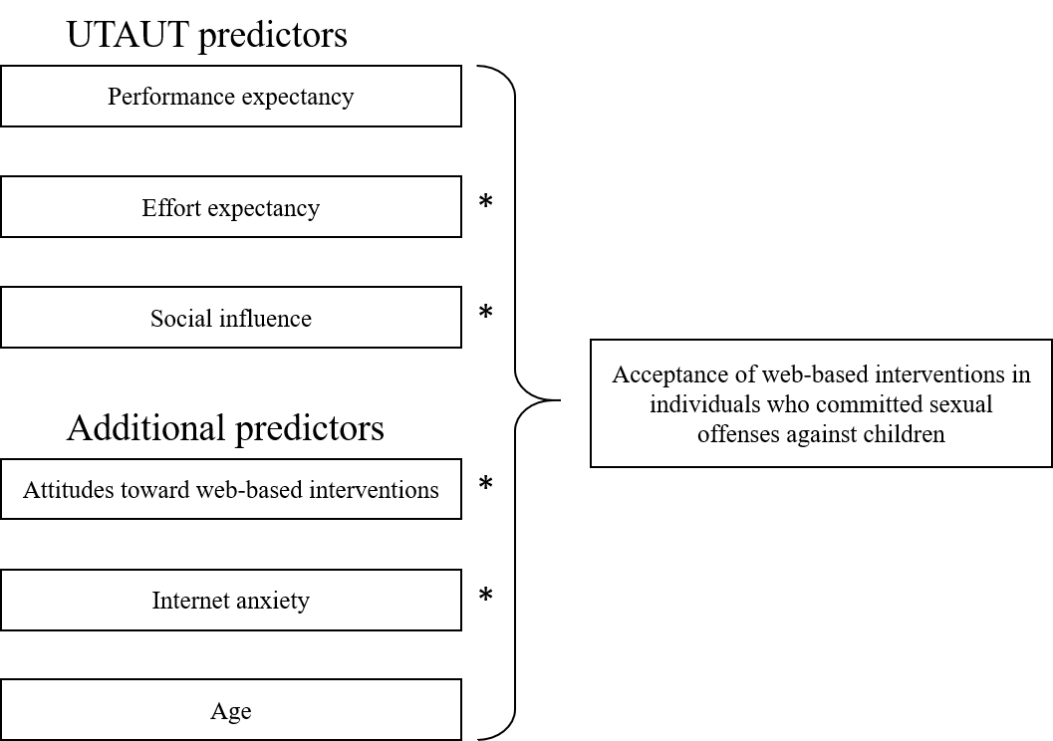
Conceptual study model with the UTAUT predictors [15] and additional variables as well as age as moderator. UTAUT: Unified Theory of Acceptance and Use of Technology. *Age as a moderator variable.

As shown above, no data exist for the specific target group of this study. Therefore, we assume that acceptance of web-based interventions for individuals who have committed sexual offenses against children follows the same mechanisms as for individuals who use web-based interventions in general psychiatry (Figure 1). As a consequence, it is expected that higher scores in PE, EE, SI, attitudes toward web-based interventions, and lower scores in internet anxiety predict higher scores in acceptance. In addition, we will examine whether age has a moderating and direct effect on acceptance.

## Methods

### Study Design and Data Collection

This cross-sectional study used data collected between March 1, 2021, and March 1, 2022, of an ongoing clinical trial to evaluate the effectiveness of the web-based intervention @myTabu [27,28]. Participants were individuals convicted of child abuse, of child sexual exploitation material use, or of both under the German Penal Code and were under community supervision. Further eligibility requirements were adulthood (18 years of age or older), a community supervision period of at least 6 months at study inclusion, internet access, no severe acute psychiatric disorder, no severe cerebro-organic disorder, and no severe cognitive impairment. For the recruiting process, research staff informed community supervisors of the clinical trial and asked them to inform eligible clients. When an eligible client was interested in the clinical trial, he or she was informed about the study by research staff in a personal interview. During the recruitment period, 118 interviews were conducted and 113 individuals agreed on taking part in the study.

### Measures

#### Sociodemographic and Criminological Data

For each participant, 1 research staff member (out of a total of 3 research staff members) collected sociodemographic and criminological data using a standardized data collection form. The written court judgment and records of the Federal Central Criminal Register were used as the primary source of information. If information was missing from these documents, corresponding information was obtained from participants. The modified Static-99, which is a version of the original Static-99 that omits victim-related variables, was assessed [29]. The Static-99 includes variables that have been found to be predictive of sexual reoffending among individuals who have previously committed a sexual offense. A higher score represents a higher risk [30]. Scores of the modified Static-99 range from 0 to 9. Information on the additional data that were coded during that process can be found in the study protocol of the @myTabu clinical trial [28].

#### Acceptance and Predictors

To measure acceptance and its predictors, the German adaptations of the UTAUT questionnaire by Baumeister et al [31] and Apolinário-Hagen et al [25] were used. These adaptations were based on the well-established UTAUT questionnaire [15] and the adaptations to the field of general psychiatry [17,32-34]. For this study, the questionnaires were modified to the context of a web-based intervention for individuals who committed sexual offenses against children based on face validity (Textbox 1, see Multimedia Appendix 1 for original German questionnaire).

**Items of the questionnaire for acceptance of technology with references to original studies; the sections that have been adapted for this study are italicized.**

Questionnaire description: Please read the following questions carefully and answer as spontaneously as possible. The following questions refer to a therapeutically guided program, which you can complete online and which supports you during your probation to avoid recidivism and to lead a crime-free life. The program consists of sessions that are unlocked weekly. In the questions, this program is called “online program.”

**Acceptance**

1. I can imagine trying an *online program* [31].
2. I can imagine using an *online program* regularly [...] [31].
3. I would recommend an *online program* to a friend [31].
4. I would be willing to pay for an *online program* [31].

**Performance expectancy**

1. Using an *online program* would *help me not to commit a further child abuse or to consume child sexual exploitation material* [31].
2. Using an *online program* would *improve my ability to live a crime-free life* [31].
3. Overall, an *online program* would help me *during my community supervision* [31].

**Effort expectancy**

1. Using an *online program* would be simple [31].
2. Using an *online program* would be an easy task for me [31].
3. An *online program* would be clear and easily comprehensible to me [31].

**Social influence**

1. People close to me would recommend me to use an *online program* [31].
2. My *community supervisor* would recommend me to use an *online program* [31].

**Attitudes toward web**-**based interventions**

1. Using the *online program* is a good idea [25].
2. Using the *online program* would be interesting [25].
3. Using the *online program* could be fun [25].
4. I would like to work with the *online program* [25].

**Internet anxiety**

1. The internet is something threatening to me [31].
2. I am afraid making an irrevocable mistake while using the internet [31].

According to the UTAUT, acceptance was operationalized as behavioral intention and was measured using 4 items. The UTAUT predictors PE and EE were measured using 3 items each and SI was measured using 2 items. Attitudes toward web-based interventions and internet anxiety were measured using 4 and 2 items, respectively. Responses were made on a 5-point Likert scale ranging from 1 (does not apply at all) to 5 (applies absolutely). McDonald ω total [35,36] were 0.59 for acceptance, 0.80 for PE, 0.81 for EE, and 0.83 for attitudes toward using web-based interventions, showing good reliability for PE, EE, and attitudes toward using web-based interventions and a poor reliability for acceptance [37]. For scales with 2 items, Spearman-Brown coefficient was calculated [38]. Spearman-Brown coefficient of SI was 0.21 and of internet anxiety was 0.65, showing a questionable reliability of internet anxiety and an unacceptable reliability for SI [37].

In addition to the above named scales, scales were measured on FC [31], planning behavior [39], and study compensation for hypotheses that were not part of this study.

### Statistical Analyses

#### Research Questions 1 and 2: Acceptance and its Predictors

Data analysis was performed using the software R (version 4.2.1; R Core Team) [40]. The mean acceptance score was calculated and its distribution was assessed. The acceptance mean score was categorized as low (1-2.34), moderate (2.35-3.67), and high (3.68-5), in accordance with previous studies [41,42].

To test for predictors of acceptance, a multiple linear regression with acceptance as the criterion was conducted. The variables PE, EE, SI, attitudes toward web-based interventions, internet anxiety, and age were included along with a moderation of age on all variables (age × variable). The predetermined α level was .05. Variables were included simultaneously in the model. For meaningful interpretation of the coefficients of the first-order terms in the presence of interactions, we mean-centered the variables prior to computation [43]. For missing items responses, the mean across the available items of each scale was calculated. There were missing items for 5 participants with a maximum of 6 missing items (mean 2.2, SD 2.17). There was no missing scale, as every participant answered at least 1 item on every scale [44]. To test for outliers, Cook distance, leverage value, and studentized deleted residuals were calculated. After correcting for coding errors, there were 23 participants who were considered outliers by the above named criteria. To test the model assumptions, we looked at linear relationships between the variables and acceptance, normality of residuals [45], homoscedasticity [46], and multicollinearity [47-49]. There were signs of nonnormality of residuals; the other analyses showed no assumption violation. Because of the outliers and the nonnormality of residuals, a bootstrap procedure was used with the number of bootstrap samples of 1000. By using bootstrapping, results are less sensitive to extreme values and thus no participant had to be excluded from the analysis [49,50].

#### Explorative Analysis

Because of the low internal consistency of SI, a multiple linear regression was conducted with acceptance as the criterion and the items of the SI scale as factors with the lowest value as reference. In addition, the predictors PE, EE, internet anxiety, attitudes toward web-based interventions, and age were included. For the SI item (asking whether the community supervisor recommends the use of a web-based intervention), values 1 and 2 were too infrequent for a statistical analysis and were thus combined into 1 category with 3. Because of missing values on SI items, 2 participants were excluded from the analysis. There were 9 outliers according to Cook distance, leverage value, and studentized deleted residuals. There were signs of nonnormality of residuals [45]; the other analyses showed no assumption violation. Therefore, a bootstrap procedure with the number of bootstrap samples of 1000 was used [49].

### Ethical Considerations

This study was conducted in accordance with the Declaration of Helsinki, was approved by the medical ethical board of the Human Medical Center Göttingen, Göttingen, Germany (16/2/20), and was preregistered on AsPredicted (107090). During study enrollment, informed consent was obtained from all participants. In the informed consent, participants agreed on the study conditions and data protection and processing. Study data were saved and deidentified by using pseudonyms for each participant. During participation, identification of each individual was only possible by the respective community supervisor. After participation, identification lists were stored separately from the study data in paper form in a safe. Individuals received monetary compensation for their participation; the compensation level was dependent on the number of sessions completed in the web-based intervention. A maximum of €120 (US $131.06; €1 is approximately US $1.2 at the start of the clinical trial) could be obtained.

## Results

### Demographic and Criminological Data

All 113 participants were male and had a median age of 38 years with a range of 20-72 (mean 40.67, SD 12.75 years). The participants had on average 1.25 previous convictions (SD 2.47). For 57.1% (64/112; 1 missing) of the participants, the present conviction was their first. For their present conviction, 38.9% (44/113) of the participants were convicted for sexual abuse of children (German Penal Code section 176 in the version in effect before July 01, 2021), 14.3% (16/113) for aggravated sexual abuse of children (German Penal Code section 176a in the version in effect before July 01, 2021), and 74.3% (84/113) for dissemination, procurement, and possession of child pornographic content (German Penal Code section 184b). Note that 28 participants had more than 1 present conviction. The mean score for the modified Static-99 was 1.87 (SD 1.19; range 0-6).

### Descriptive Data of Acceptance and Predictors

The mean (SD) acceptance level in this study was 3.78 (SD 0.66). The distribution of acceptance is negatively skewed with 2.7% (3/113) of the participants indicating low, 36.3% (41/113) moderate, and 61.1% (69/113) high acceptance. The mean score of PE was 4.08 (SD 0.77), of EE was 4.10 (SD 0.67), of SI was 3.88 (SD 0.81), of attitudes toward web-based interventions was 4.15 (SD 0.63), and of internet anxiety was 2.02 (SD 0.93).

### Prediction of Acceptance

According to the *F* test (*F*_11,101_=9.055), the variables in the regression model explained 41.2% of the variance of acceptance (*R*^2^=0.412; *P*<.001; Table 1). With a regression coefficient of *B*=0.398 (95% CI 0.16-0.64; *P*=.01) for attitudes toward web-based interventions and *B*=0.184 (95% CI 0.03-0.38; *P*=.04) for SI, there were significant linear effects of both variables on acceptance. The other variables did not predict acceptance above the effects of attitudes toward web-based interventions and SI (all *P*>.05). There was no moderating effect of age on any variables (all *P*>.05).

**Table 1:** Regression results using bootstrapping with acceptance as the criterion (N=113)^a^.

Variables	*B* (SE)	95% CI	*T*	*P* value
Intercept	3.77 (0.056)	3.64 to 3.87	67.129	<.001
PE^b^	0.03 (0.093)	–0.15 to 0.23	0.332	.74
EE^c^	0.09 (0.104)	–0.13 to 0.29	0.882	.38
SI^d^	0.18 (0.087)	0.03 to 0.38	2.109	.04
Attitudes^e^	0.40 (0.118)	0.16 to 0.64	3.372	.01
Anxiety^f^	–0.03 (0.068)	–0.19 to 0.09	–0.462	.64
Age	0.01 (0.005)	0.01 to 0.01	1.036	.30
PE × age	–0.01 (0.008)	–0.03 to 0.00	–1.543	.13
EE × age	–0.01 (0.009)	–0.03 to 0.00	–1.435	.15
Attitudes × age	–0.003 (0.010)	–0.02 to 0.01	–0.340	.73
SI × age	0.01 (0.007)	–0.001 to 0.03	1.630	.11
Anxiety × age	–0.01 (0.006)	–0.02 to 0.00	–1.099	.27

^a^*R*^2^=0.412; *P*<.001; 95% CI 0.20-0.48.

^b^PE: performance expectancy.

^c^EE: effort expectancy.

^d^SI: social influence.

^e^Attitudes: attitudes toward web-based interventions.

^f^Anxiety: internet anxiety.

### Explorative Analysis

The mean (SD) score for the item of the SI scale asking whether people close to the participant recommend the use of a web-based intervention was 3.24 (SD 1.20). The mean (SD) score for the item of the SI scale asking whether the community supervisor recommends the use of a web-based intervention was 4.49 (SD 0.80).

The variables in the regression model explained 35.95% of the variance of acceptance (*F*_11,99_=6.273; *R*^2^=0.3595; *P*<.001). Attitudes toward web-based intervention (*B*=0.331; 95% CI 0.09-0.55; *P*=.01) and the perceived opinion of people close to the participant significantly predicted acceptance. Participants who indicated a score of 5 (*B*=0.455; 95% CI 0.07-0.96; *P*=.04) or 4 (*B*=0.502; 95% CI 0.17-0.89; *P*=.01) had significantly higher acceptance than participants who indicated a score of 1. There was no significant effect for participants who indicated a score of 3 (*B*=0.340; 95% CI 0.01-0.72; *P*=.07) or 2 (*B*=0.182; 95% CI –0.31 to 0.60; *P*=.42) in comparison to participants who indicated a score of 1. PE (*B*=0.108; 95% CI –0.08 to 0.28; *P*=.24), EE (*B*=0.106; 95% CI –0.12 to 0.29; *P*=.30), internet anxiety (*B*=–0.06; 95% CI –0.22 to 0.09; *P*=.46), age (*B*=0.006; 95% CI –0.005 to 0.01; *P*=.22), and perceived opinion of the community supervisor, when scored 4 (*B*=–0.05; 95% CI –0.47 to 0.25; *P*=.79) or 5 (*B*=0.094; 95% CI –0.18 to 0.42; *P*=.55) in comparison to lower or equal to 3, did not significantly predict acceptance.

## Discussion

### Principal Results

This study examined for the first time the acceptance of web-based interventions and variables predicting it among individuals who committed sexual offenses against children. For the majority of participants, the acceptance of web-based interventions was high. Persons with higher scores in SI and attitudes toward web-based interventions showed significantly higher acceptance. In contrast to expectations, the other predictors of the UTAUT, PE and EE, as well as internet anxiety and age did not predict acceptance. An explorative analysis of the 2 items comprising the SI scale revealed that the belief that people close to the participant would recommend the use of a web-based intervention predicts acceptance but the same is not true for the belief that the community supervisors would recommend the use thereof.

### Comparison With Prior Work

In comparison to prior work from general psychiatry, the average acceptance was higher in this study with a smaller variance. In the secondary analysis from Philippi et al [16], in which results from 10 original studies were included, the mean acceptance for male participants was low to moderate (mean 2.68, SD 1.12). One explanation for the high acceptance scores in this study may be that it is difficult for individuals who committed sexual offenses against children to find f2f therapy [5]. Another explanation for the divergent acceptance levels may be differences in the sample selection. A common recruiting method to contact specific target groups in the studies incorporated in the secondary analysis by Philippi et al [16] was to contact patients directly in clinics [31,32,41], for example by recruiting in the waiting rooms [14]. To protect the identity of clients, recruitment in this study involved collaboration with community supervisors. Thus, the research staff did not contact eligible clients directly but instead asked the community supervisors to inform eligible clients of the study. Through this approach, it is likely that some clients were never informed about the study because they were considered unsuitable by the community supervisor. In addition, clients who declined after first being informed by the community supervisor never met with research staff. Further, the participants in this study all agreed to participate in an evaluation study for a web-based intervention. The aim of most of the studies incorporated in the secondary analysis by Philippi et al [16] was to test acceptance-facilitating interventions and participants were not given access to a web-based intervention. Thus, it is likely that the results are based on a selected group of individuals in community supervision, which may not be representative of individuals who committed sexual offenses against children in general. This explanation is in line with the study by Lin et al [22], who recruited participants by sending invitations to individuals who had earlier expressed interest in participating in an evaluation study on a web-based intervention and (thus) assessed comparatively high acceptance (mean 3.44, SD 0.89; values were divided by 4 to match the scale used in this study). Despite this potential bias, the results of this study show that there exists a group of individuals who committed sexual offenses against children in community supervision that has high acceptance of web-based interventions.

Participants in this study rated web-based interventions as more helpful (PE), easier to use (EE), more enjoyable (attitudes toward web-based interventions), and perceived that their social surroundings would recommend the use of web-based interventions more (SI) than did participants in studies of web-based interventions in general psychiatry. All of these predictors are positively correlated with acceptance [16,25]. As mentioned above, this positive view of web-based interventions can be partly explained by the selection of the sample. In comparison, internet anxiety, which has a negative correlation with acceptance, was found to be slightly lower in studies from general psychiatry [16]. For individuals who were not convicted for a crime using the internet, this result could be understood when considering that internet anxiety is negatively correlated with internet use [51] and convicted individuals often lack the skills and resources to use the internet or specific technologies [52]. Although the internet anxiety levels in this study cannot be considered as high, a lack of experience with the internet could be a more important issue for individuals who committed sexual offenses against children compared to individuals who have not been convicted of a crime.

The proportion of explained variance of 41.2% in the regression model can be considered as high according to the Cohen criteria [53]. However, this proportion is lower than in other UTAUT studies, where UTAUT predictors explained for example 57% to 63% of the variance of acceptance [21,54,55]. This could mean that, for individuals who have committed a crime, further predictors are relevant that have to be investigated in order to fully understand the acceptance of web-based interventions. In this study, to test our hypotheses, only selected variables that were replicated in previous studies on web-based interventions in general psychiatry were examined for their prediction on acceptance [16,20,25]. In studies on web-based interventions in general psychiatry, further variables that have been investigated include, among others, perceived reliability [56] and perceived privacy risks [54]. Next to these variables, those that predict the treatment motivation for f2f therapy in individuals who committed sexual offenses against children, for example, antisocial personality disorder, might also be relevant for web-based interventions [57]. In addition, web-based interventions are becoming more common in general mental health care [58] and are increasingly being developed for individuals who committed sexual offenses against children [8]. Therefore, it is likely that an increasing number of individuals have some experience with web-based interventions which could have a direct or moderating effect on acceptance [15]. These and other variables could be important when explaining the variance of acceptance of web-based interventions in individuals who committed sexual offenses against children.

In previous studies, it has been repeatedly shown that the original UTAUT predictors PE, EE, and SI are predictive of acceptance [16,59]. In this study, against our expectations, the predictive effect of PE and EE could not be replicated for individuals who had committed sexual crimes against children. In contrast, SI and attitudes toward web-based interventions were significant predictors of acceptance. Attitudes toward web-based interventions was also found to be a strong predictor of acceptance in other subject groups and was equally as strong as PE [20,25]. The importance of attitudes for acceptance may be related to the fact that the participants in this study most likely had no specific knowledge or experience with web-based interventions at the time they answered the UTAUT items. In this state of indecision, positive attitudes might be more important than cognitive beliefs about the web-based intervention. That could be a reason why the hypothesis that PE and EE are predictive for acceptance was refuted in this study [25].

The significant prediction by SI of acceptance could be explained by the fact that participants are influenced by how other people, especially their community supervisors, evaluate their community supervision time. However, in the exploratory analysis, it was found that the perceived higher opinion of people close to the participant but not the perceived lower or higher opinion of the community supervisor significantly predicted higher acceptance. A reason why the perceived opinion of the community supervisor was not predictive could be that most participants rated the opinion of their community supervisor as high. This may be because community supervisors who did not support web-based interventions may have not informed their clients. Thus, the results of this explorative analysis could imply that especially in a situation where the community supervisors support a web-based intervention, the opinion of people close to the participant predicts acceptance.

In previous studies, it was found that lower age predicted higher acceptance [19-21] and that the effect of PE, EE, and SI was moderated by age [15]. In this study, however, no direct or moderating effect of age could be observed in individuals who committed sexual offenses against children. This is in line with studies by Philippi et al [16] and Lin et al [22], who also could not replicate a direct or moderating effect of age.

### Limitations

The first limitation arises from the sample selection. As mentioned above, participants were preselected by community supervisors and the participants were persons who already agreed to take part in a web-based intervention study. Because of that, it is not clear if and how representative the sample is of individuals who committed sexual offenses against children and who are presently in community supervision and thus how generalizable the results of this study are.

The second limitation could have resulted from the preselection. The variances in this study are low, which could be an indicator that the sample variance is lower than the actual population variance. Because of that restriction of variance, the statistical power to detect interactions is reduced [43].

The third limitation is that the scales acceptance, SI and internet anxiety show low reliability. For this study, we used the well-established UTAUT questionnaire [15] and adaptations used in the field of general psychiatry [17,32-34]. The questionnaire for this study was based as closely as possible on this format. However, some aspects of general psychiatry may not be transferable to the context of this study. For example, the acceptance scale includes an item asking whether participants would recommend a web-based intervention to a friend. For individuals convicted of a crime, shame and the need to hide the conviction from those close to them could be relevant aspects that might influence the answer to this item [60].

The fourth limitation is that the questionnaire was completed in the presence of the research staff. Therefore, the participants might have answered in a socially desirable manner, for example, to appear cooperative toward the study.

### Future Directions and Clinical Implications

Future research should examine the predictive power of further variables that go beyond the UTAUT model. Variables that are possibly relevant are described in the previous section (eg, perceived reliability, antisocial personality disorder, and experience with web-based interventions). To increase acceptance, it should be tested whether acceptance-facilitating interventions, that highlight the positive aspects of using a web-based intervention (attitudes toward web-based interventions) and address reasons why the potential users assume that people close to them may not be in favor of them (SI) are especially effective. To that end, it should be investigated whether there are differences in acceptance depending on the characteristics of the potential users (eg, conviction type and the number of previous convictions). By doing that, acceptance-facilitating intervention could be tailored to the specific needs of the potential participants and may be more effective [31]. Further, research should look at the actual use of web-based interventions and test whether acceptance, as hypothesized by UTAUT, can predict factors like satisfaction or need fulfillment [61] and the actual use of a web-based intervention.

### Conclusions

This study is the first to analyze the acceptance of web-based intervention in individuals who committed sexual offenses against children. In this study the acceptance levels of the majority of participants were high. The perceived opinion of the social contacts, as well as, the attitudes toward web-based interventions was important in predicting acceptance. To increase acceptance, it may be important to incorporate these predictors when designing acceptance-facilitating interventions.

## Appendix

Multimedia Appendix 1 German questionnaire items used in this study with references to original studies.

## Abbreviations

EE: effort expectancy
FC: facilitating conditions
f2f: face-to-face
PE: performance expectancy
SI: social influence
UTAUT: Unified Theory of Acceptance and Use of Technology

## Data Availability

The data sets analyzed during this study are not publicly available due to their high sensitivity (eg, conviction of sexual abuse of children), the lack of possibility for complete anonymization, and the fact that the @myTabu clinical trial is ongoing but are available from the corresponding author on reasonable request.
