# Supplementary material for: Acceptance of a Web-Based Intervention in Individuals Who Committed Sexual Offenses Against Children: Cross-Sectional Study
Source: JMIR Form Res. 2024 Jan 26;8:e48880. doi: 10.2196/48880 (PMC10858427; doi:10.2196/48880)
Supplement: Multimedia Appendix 1 [file formative_v8i1e48880_app1.docx]

| Scale | Items |
| --- | --- |
| Behavioral intention | 1. Ich kann mir grundsätzlich vorstellen ein Online-Programm auszuprobieren. [31] 2. Ich würde ein Online-Programm regelmäßig nutzen. [31] 3. Ich würde einer Freundin oder einem Freund ohne zu zögern ein Online-Programm empfehlen. [31] 4. Ich wäre bereit, für ein Online-Programm Geld zu bezahlen. [31] |
| Performance expectancy | 1. Die Nutzung eines Online-Programms würde mir helfen, keinen erneuten Kindesmissbrauch zu begehen oder Missbrauchsabbildungen von Kindern anzuschauen. [31] 2. Die Nutzung eines Online-Programms würde meine Fähigkeiten, ein straffreies Leben zu führen, verbessern. [31] 3. Insgesamt würde mir ein Online-Programm während meiner Bewährungszeit bzw. Führungsaufsicht helfen. [31] |
| Effort expectancy | 1. Die Nutzung eines Online-Programms würde einfach sein. [31] 2. Der Umgang mit einem Online-Programm würde mir leicht fallen. [31] 3. Die Nutzung eines Online-Programms würde für mich klar und verständlich sein. [31] |
| Social influence | 1. Mir nahestehende Personen würden mir zur Teilnahme an einem Online-Programm raten. [31] 2. Mein Bewährungshelfer bzw. meine Bewährungshelferin würde mir zur Teilnahme an einem Online-Programm raten. [31] |
| Facilitating conditions | 1. Ich verfüge über die notwendigen technischen Voraussetzungen, um ein Online-Programm zu nutzen. [31] 2. Bei technischen Problemen mit einem Online-Programm würde ich Unterstützung bekommen. [31] |
| Internet anxiety | 1. Das Internet hat für mich etwas Bedrohliches. [31] 2. Bei der Nutzung des Internets habe ich Angst, einen nicht behebbaren Fehler zu begehen. [31] |
| Attitude toward web–based interventions | 1. Es ist eine gute Idee, ein Online-Programm zu nutzen. [25] 2. Es wäre interessant, ein Online-Programm zu nutzen. [25] 3. Die Nutzung eines Online-Programms könnte Spaß machen. [25] 4. Ich würde es mögen, mit einem Online-Programm zur Unterstützung zu arbeiten. [25] |
| Planning | 1. Ich habe bereits konkret geplant, wann ich Sitzungen von @myTabu bearbeiten werde. [39] 2. Ich habe bereits konkret geplant, wie oft ich in der Woche an Sitzungen von @myTabu arbeiten werde. [39] 3. Ich habe bereits konkret geplant, wie ich trotz meiner anderen Verpflichtungen und Interessen Sitzungen von @myTabu bearbeiten werde. [39] 4. Ich habe bereits konkret geplant, wie ich weiterhin Sitzungen von @myTabu bearbeiten werde, auch wenn einmal etwas dazwischenkommt. [39] |
| Incentives | 1. Für @myTabu Geld zu erhalten, motiviert mich an Sitzungen teilzunehmen. 2. Innerhalb von @myTabu Auszeichnungen zu erhalten, motiviert mich an Sitzungen teilzunehmen. |
